# Echocardiographic Evaluation of Changes in Cardiac Hemodynamics and Loading Conditions after Transthoracic Minimally Invasive Device Closure of Atrial Septal Defect

**DOI:** 10.1371/journal.pone.0128475

**Published:** 2015-07-06

**Authors:** Qiang Chen, Xu-Dong Sun, Hua Cao, Gui-Can Zhang, Liang-Wan Chen, Yun-Nan Hu

**Affiliations:** 1 Department of Cardiovascular Surgery, Union Hospital, Fujian Medical University, Fuzhou, P. R. China; 2 Department of Cardiology, Union Hospital, Fujian Medical University, Fuzhou, P. R. China; Temple University, UNITED STATES

## Abstract

**Purpose:**

To evaluate transthoracic minimally invasive device closure of atrial septal defects by performing transthoracic echocardiography to measure changes in cardiac hemodynamics and loading conditions.

**Methods:**

Between January 2012 and December 2012, we performed transthoracic minimally invasive device closure of atrial septal defects in 95 patients with secundum atrial septal defects (ASD), and performed transthoracic echocardiography to measure blood flow velocities at the tricuspid valve orifice and at the pulmonary valve orifice, sizes of the left and right atria and ventricles, right ventricular fractional area change, right ventricular Tei index, three-dimensional right ventricular ejection fraction, tricuspid annular plane systolic excursion and left ventricular ejection fractions before the procedure and 1 week, 3 months, and 1 year post-procedure.

**Results:**

Varying degrees of improvement were observed post-procedure at later time points. The maximum blood flow velocity at the pulmonary valve orifice, mean flow velocity, velocity-time integral, and A peak and E peak blood flow velocity at the tricuspid valve orifice decreased significantly post-procedure (*P*<0.05). In 3 months and 1 year’s follow-up, the inner diameter of the middle portion of the pulmonary artery, and diameters of the right atrium and right ventricle decreased significantly post-procedure (*P*<0.05). The diameters of the left atrium and left ventricle increased after the procedure (*P*<0.05). One week after the procedure, the right ventricular fractional area change, three-dimensional right ventricular ejection fraction, right ventricular Tei index and tricuspid annular plane systolic excursion had significantly reduced compared with the preoperative data (P<0.05). While these four parameters were still decreased at the 3 months and at 1 year’s follow-up, but the differences were not statistically significant compared with the 1 week’s postoperative data (P>0.05). One week post-procedure, left ventricular ejection fraction had not changed significantly, but at 3 months and at 1 year post-procedure, left ejection fraction had increased significantly compared with the preoperative data (P<0.05).

**Conclusion:**

Echocardiographic evaluation has demonstrated that cardiac hemodynamics and loading conditions improved significantly after transthoracic minimally invasive device closure of atrial septal defects.

## Introduction

Atrial septal defect (ASD) is a common congenital heart disease, the treatment of which includes intracardiac repair under direct vision with extracorporeal circulation, or percutaneous closure. [1] In recent years, we have devised a technique that shares characteristics of percutaneous closure and open surgical repair. With this technique, transthoracic minimally invasive device closure of atrial septal defects, we have achieved satisfactory outcomes. [2–4] Some reports describe the application of ultrasonography to evaluate clinical outcome after using the Amplatzer atrial septal occluder [5–7]; however, there have been few published reports on ultrasonographic evaluation of outcomes after transthoracic minimally invasive device closure of atrial septal defect. In the current study, we measured preoperative and postoperative hemodynamics, changes in the size of each heart chamber, right ventricular fractional area change, right ventricular Tei index, three-dimensional right ventricular ejection fraction, tricuspid annular plane systolic excursion and changes in left ventricular ejection fraction, to evaluate the effectiveness of transthoracic minimally invasive device closure of atrial septal defect.

## Materials and Methods

The present study was approved by the ethics committee of our university, and adhered to the tenets of the Declaration of Helsinki. Written informed consent was obtained from the patients or parents of the patients.

### 1 Clinical data

Ninety-five patients who underwent transthoracic minimally invasive device closure of atrial septal defect in our hospital between January 2012 and December 2012 were selected for the current study. There were 42 males and 53 females, with a mean age of 18.2±16.6 years (range, 3–52 years), and a mean body weight of 28.5±15.1 kg (range, 15–70 kg). These patients chose intraoperative device closure for treatment of their atrial septal defects. The indications for our procedure were the same as those used for surgical or percutaneous closure, which included a hemodynamically significant left-to-right shunt, significant chamber enlargement, mild-to-moderate or severe pulmonary hypertension despite medical therapy, a history of infective endocarditis, or any combination of the above indications. Those with other coexisting cardiac anomalies were excluded from our study. [4] Using echocardiography, all patients were diagnosed with secundum atrial septal defect without other associated intracardiac malformations. We have used different transthoracic echocardiographic measurement views to assess the diameter of the defect, and chose the largest measured diameter as our pre-closure measurement. Measurement of right ventricular systolic pressure revealed that thirty-two patients had associated mild-to-moderate pulmonary hypertension. In these patients, pulmonary artery systolic pressure was 30 to 60 mm Hg. No patient had severe pulmonary hypertension. The ratio of pulmonary-to-systemic blood flow (Qp/Qs) was greater than 1.5 in all patients.

### 2 Surgical procedure

**Fig 1:**
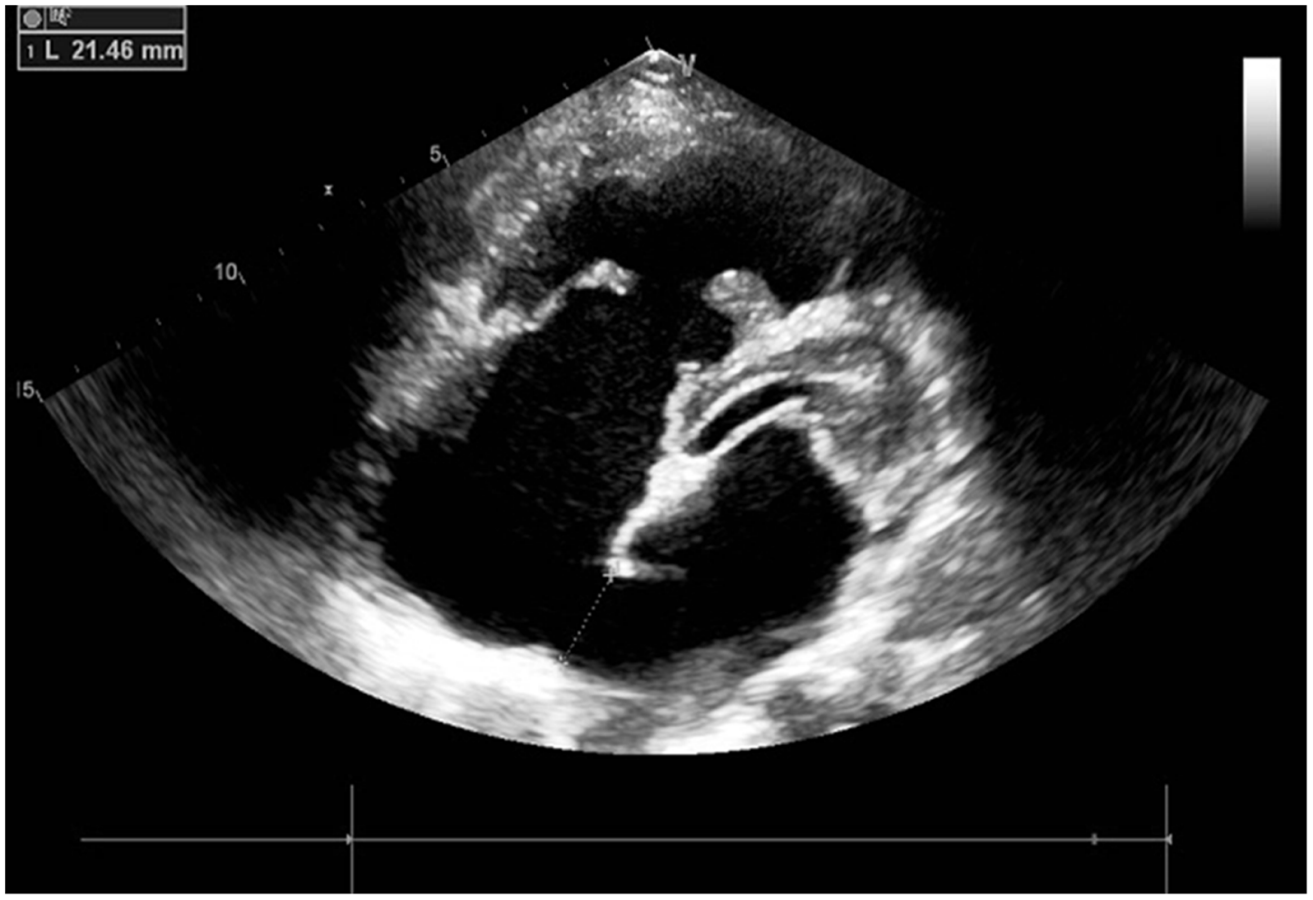
The ASD image shown by TTE.

**Fig 2:**
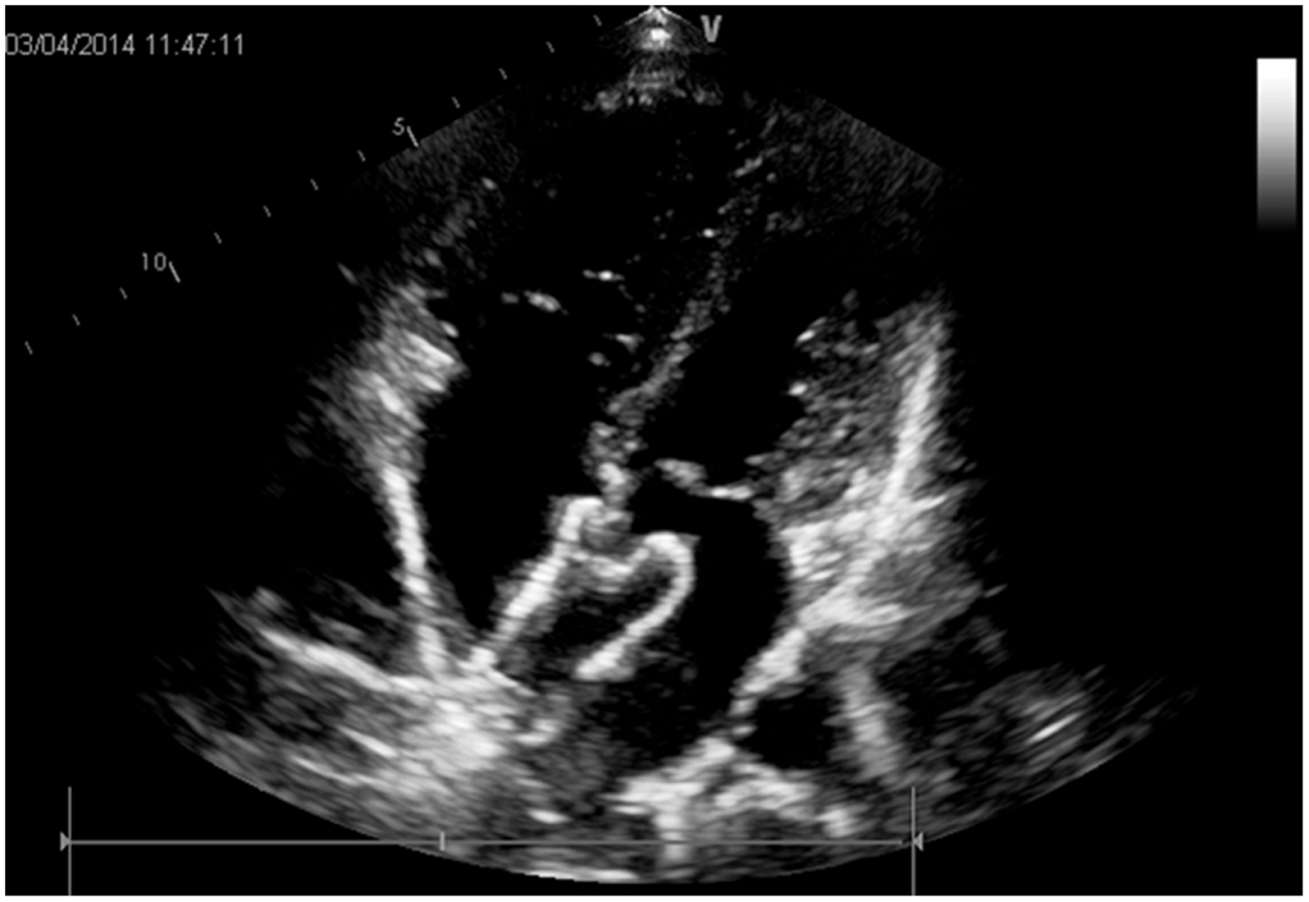
Final image shown after both discs were deployed.

The locations, sizes, and margins of atrial septal defects were identified using transthoracic echocardiography prior to the procedure for screening cases for the study. (Fig 1) [5–7] An appropriately sized occluder (which was manufactured at Dong Guan Ke Wei Medical Apparatus Co. Ltd. of China) was selected according to the preoperative echocardiography measurement, and was placed in the delivery sheath after soaking in heparin saline. During the procedure, under the guidance of a parasternal ultrasonographic 4-chamber view, the delivery sheath was placed into the left atrium through the ASD. Then, via the sheath, the occluder inside the left atrium was opened, and it was then pulled back tightly. Thereafter, the occluder inside the right atrium was opened. (Fig 2) After the margin of the ASD was clamped with the bilateral occluder, echocardiography was performed again to confirm the tightness of the occluder, making sure that there was no residual shunt and that there was no adverse impact on the structure and function of the superior vena cava, inferior vena cava, the pulmonary veins, coronary sinus orifices, mitral valve, or tricuspid valve. [2–4]

### 3 Transthoracic echocardiographic measurement views and contents

(1) The short axial view of the great vessels was used to measure the maximum systolic blood flow velocity at the pulmonary artery orifice, the mean blood flow velocity, the velocity-time integral, and the inner diameter of the middle portion of the pulmonary artery. (2) Apical four-chamber view was used to measure the diastolic E peak and A peak blood flow velocities at the tricuspid valve orifice. The end-diastolic length and width of the right ventricle, the end-systolic and end-diastolic lengths of the left ventricle, and the end-systolic lengths and widths of the right atrium and the left atrium were also measured. (3) The short axial view of the left ventricle was used to measure the end-systolic and end-diastolic anteroposterior diameters and widths of the left ventricle at the level of the chordae tendineae. (4) The parasternal long axis view of the left ventricle was used to measure the end-diastolic anteroposterior diameter of the middle portion of the right ventricle. (5) The left ventricular ejection fractions were calculated.

### 4 Right ventricular fractional area change (RVFAC)

RVFAC was defined using the formula (end-diastolic area—end-systolic area)/end-diastolic area×100%.[8] Using the apical 4-chamber view, endocardial borders of the right ventricular free wall and septum were traced from base to apex, and the respective right ventricular areas were determined from the average of 3 measurements. End-diastole was identified by the onset of the R wave from a simultaneously recorded electrocardiogram, whereas end-systole was regarded as the smallest right ventricular cavity size before tricuspid valve opening.

### 5 RT-3DE images

All RT-3DE images were processed offline using a dedicated analysis software package (4D Echo-View, TomTec Imaging Systems, Munich, Germany). After manual selection of the central reference points of the tricuspid and mitral valve annuli and of the LV apex, the software visualized the RV cavity by the coronal, sagittal, and frontal cut planes automatically obtained from the 3D data set both at end-systole and at end-diastole. After manual tracing of the endocardial borders, on each of the 3 cut planes at both end-diastole and end-systole, the software automatically detects the RV surfaces throughout the cardiac cycle. Manual correction to adjust the endocardial contours in each frame prior to quantification was performed when needed. RV volumes were computed throughout the cardiac cycle, from which RV end-diastolic volume (RVEDV) and RV end-systolic volume (RVESV) were obtained as maximums and minimums, respectively. RV ejection fraction (RVEF) were then measured as the difference and as the percentage change of the volumes, respectively. [9]

### 6 The RV Tei index

The RV Tei index is defined as (A—B)/B, where A is time interval between the end and onset of tricuspid annular diastolic velocity and B is the duration of tricuspid annular systolic velocity (or RV ejection time). And The RV Tei index were determined from the average of 3 measurements. [10,11]

### 7 The tricuspid annular plane excursion(TAPSE)

TAPSE was recorded from the apical four-chamber view. The M-mode cursor was placed at the junction of the RV free wall and the tricuspid valve in such a way that the tricuspid annulus moved along the M-mode line and the maximal longitudinal shortening and relaxation of the RV free wall was recorded. The amplitude of the excursion of tricuspid annulus from the base toward the apex in systole was defined as TAPSE. [12]

### 8 Measured times

The indicators outlined above were measured with transthoracic echocardiography before the procedure, and 1 week, 3 months, and 1 year after the procedure for all patients.

### 9 Statistical analysis

Continuous data were presented as mean± standard deviation and range. Differences time periods of datas were compared with the analysis of variance with repeated measurement data, comparing the two using Tukey HSD test. A *P* value of <0.05 was defined as statistically significant.

## Results

All patients underwent successful closure of an ASD. The mean diameter of the defect was 18.9±11.8 mm (range, 10–38 mm) and the size of the occluder was 21.3±13.6 mm (range, 14–44 mm). No significant residual shunt or device dislodgement occurred, nor did any other major complication occur during the follow-up period. The Qp/Qs ratio was significantly improved in most patients.

Table 1 shows changes in blood flow velocity at the pulmonary valve orifice and the tricuspid valve orifice. The data demonstrate that the systolic V_max_, V_mean_, and VTI at the pulmonary valve orifice decreased significantly after the procedure (*P*<0.05). In addition, E peak and A peak blood flow velocity at the tricuspid valve orifice were reduced significantly after the procedure (*P*<0.05).

**Table 1:** Changes of blood flow velocities at the pulmonary valve orifice and the tricuspid valve orifice.

Item	Preoperative	1 week after procedure	3 months after procedure	1 year after procedure
**V_max_ (cm/s)**	143.5±23.6	102.1±18.5*	95.2±12.2*	85.4±11.7*
**V_mean_ (cm/s)**	100.2±21.2	78.3±15.6*	62.5±11.1*	60.8±10.3*
**VTI (cm)**	32.1±6.5	23.1±6.4*	22.6±5.4*	21.4±6.1*
**E Peak (cm/s)**	93.2±14.8	65.8±13.4*	59.5±12.2*	51.8±13.5*
**A Peak (cm/s)**	65.9±15.2	50.1±12.5*	40.5±10.3*	35.6±9.1*

* Different from Preoperative (*P*<0.05)

Table 2 shows changes in the size and the function of the right heart before and after ASD closure. One week after the procedure, the end-systolic length and width of the right atrium, the end-diastolic anteroposterior diameter of the right ventricle, the inner diameter of the middle portion of the pulmonary artery, the length and width of the right ventricle had decreased, but the differences were not statistically significant (*P*>0.05). At 3 months and at 1 year after ASD closure, the end-systolic length and width of the right atrium, end-diastolic anteroposterior diameter, length and width of the right ventricle, and the inner diameter of the middle portion of the pulmonary artery were significantly reduced compared with the preoperative data (P<0.05).

**Table 2:** Changes in right heart size before and after atrial septal defect closure.

Item	Preoperative	1 week after procedure	3 months after procedure	1 year after procedure
**End-systolic length of the right atrium (mm)**	58.6±7.5	54.1±7.3	47.4±6.7*	45.4±5.9*
**End-systolic width of the right atrium (mm)**	50.1±8.2	47.7±8.5	38.2±7.3*	37.5±6.6*
**End-diastolic anteroposterior diameter of the right ventricle (mm)**	34.5±6.5	28.4±5.8	25.6±5.7	23.4±5.5
**End-diastolic length of the right ventricle (mm)**	70.6±11.2	67.4±11.4	61.5±11.6*	60.8±12.4*
**End-diastolic width of the right ventricle (mm)**	45.7±10.5	41.3±10.7	35.5±8.8*	32.7±9.6*
**Inner diameter of the middle portion of the pulmonary artery (mm)**	26.5±4.3	24.3±3.5	23.5±3.8*	21.6±5.4*
**RVFAC(%)**	47.1±3.2	43.3±2.5*	41.1±2.9*	40.5±3.5*
**RV-Tei**	0.48±0.07	0.39±0.05*	0.33±0.04*	0.31±0.05*
**3D-RVEF(%)**	62.1±3.6	53.8±2.5*	51.9±3.1*	50.5±2.7*
**TAPSE(mm)**	23.1±3.6	20.9±3.7*	20.1±2.5*	19.5±3.2*

* Different from Preoperative (*P*<0.05)

One week after the procedure, the right ventricular fractional area change, three-dimensional right ventricular ejection fraction, right ventricular Tei index and tricuspid annular plane systolic excursio had significantly reduced compared with the preoperative data (P<0.05). While these four parameters were still decreased at the 3 months and at 1 year’s follow-up, but the differences were not statistically significant compared with the 1 week’s postoperative data (P>0.05).

Table 3 shows changes in the size of the left ventricle before and after ASD closure. One week after the procedure, the end-systolic length and width of the left atrium, the end-systolic length and the end-systolic and end-diastolic anteroposterior diameters of the left ventricle were increased, but the differences were not significant (*P*>0.05); the end-systolic width of the left ventricle were increased significantly compared with the preoperative data (*P*<0.05). At 3 months and at 1 year after the procedure, the end-systolic length and width of the left atrium and the end-systolic and end-diastolic anteroposterior diameters, and the width and length of the left ventricle had increased significantly (*P*<0.05). In 3 months and 1 year’s follow-up, the left ventricular ejection fraction had increased significantly compared with the preoperative data (*P*<0.05).

**Table 3:** Changes in left heart size before and after closure of atrial septal defect.

Item	Preoperative	1 week after procedure	3 months after procedure	1 year after procedure
**End-systolic length of the left atrium (mm)**	44.3±6.5	44.8±7.1	46.4±6.3*	46.5±5.8*
**End-systolic width of the left atrium (mm)**	32.2±3.5	32.7±4.5	37.2±4.3*	37.5±4.2*
**End-systolic anteroposterior diameter of the left ventricle (mm)**	25.1±6.5	26.4±5.7	27.3±5.8*	29.4±6.2*
**End-systolic width of the left ventricle (mm)**	27.6±4.2	29.4±5.4*	31.5±5.6*	32.6±5.7*
**End-systolic length of the left ventricle (mm)**	47.7±7.5	49.4±6.7	51.1±7.8*	51.7±8.6*
**End-diastolic anteroposterior diameter of the left ventricle (mm)**	40.4±4.2	43.3±5.5	45.5±5.8*	46.6±5.3*
**End-diastolic width of the left ventricle (mm)**	41.7±3.5	43.3±5.7	45.6±5.8*	46.7±5.6*
**End-diastolic length of the left ventricle (mm)**	64.5±8.3	67.3±8.5	69.5±7.8*	69.6±8.6*
**Left ventricular ejection fraction (%)**	52.2±5.1	58.3±4.5	60.4±4.6*	62.1±5.3*

* Different from Preoperative (*P*<0.05)

## Discussion

Patients with ASD are usually asymptomatic and thus, most patients with this condition could wait to undergo elective intracardiac repair under direct vision with extracorporeal circulation, or undergo percutaneous closure. In recent years, many surgeons have tried to repair ASDs using transthoracic minimally invasive device closure, and have achieved satisfactory clinical outcomes. [2–4,13,14] This technique can completely avoid extracorporeal circulation, and the small incision (3–5cm) in the right anterior chest wall is not objectionable from an aesthetic standpoint. In addition, both medical expenses and use of resources may be less with this technique than with surgical closure, and there is no need for X-rays or other expensive equipment. [2–4] We believe that transthoracic minimally invasive device closure may be the treatment of choice for patients with ASD in some underdeveloped countries with scant medical and economic resources, and for patients with peripheral vascular conditions for which catheter closure would not be suitable.

In our previous reports, we demonstrated that transthoracic echocardiography can play an important role in the surgical procedure. Some investigators have reported that transesophageal echocardiography and real-time three-dimensional ultrasound are more accurate than transthoracic echocardiography; [15] however, we believe that transthoracic echocardiography can be used for preoperative patient selection and intraoperative occluder placement with the assistance of an experienced ultrasonographer. [16–19] There have been many reports of follow-up of patients undergoing percutaneous ASD closure, most of these reports are concerned with residual shunting, occluder displacement, or thrombotic events. [20,21] Other investigators have reported results of echocardiography during postoperative follow-up of patients who have undergone percutaneous closure of ASD. [22–24] Eroglu et al. concluded that percutaneous closure results in rapid remodeling and normalization of RV deformation, and the major geometrical and deformational changes are completed in 24 hours. [25] Takaya et al. reported that cardiac remodeling and exercise capacity could be improved over a long-term period after transcatheter closure of ASD in middle-aged and elderly patients. [26] There were differences in the time course of improvement between cardiac remodeling and exercise capacity in those patients. Kaya and his colleagues investigated the intermediate-term effects of transcatheter ASD closure on cardiac remodeling in children and adult patients. They concluded that transcatheter ASD closure leads to a significant improvement in clinical status and heart cavity dimensions in adults and children, as revealed by intermediate-term follow-up evaluation. [27]

Because a right-to-left shunt occurs in patients with ASD, volume loading of the right heart is increased, which leads to increased blood flow velocity at the tricuspid valve orifice and the pulmonary valve orifice, and an enlarged right atrium and right ventricle, finally resulting in high-volume loading and a hyperkinetic circulatory state. Ağaç et al. reported that closure of ASD using Amplatzer devices led to a decrease in the right heart chamber size, tissue Doppler-derived tricuspid annular velocities, and tricuspid annular plane systolic excursion in the early period. [28] Other reports have focused on right ventricular remodeling, they achieved results in rapid normalization of RV volume overload and improvement of RV function after transcatheter closure of an ASD. [29,30]

In the current study, we observed that after ASD closure, the real-time right-to-left shunt disappeared, the diastolic blood flow velocity at the tricuspid valve orifice and the systolic blood flow velocity at the pulmonary valve orifice decreased, and hemodynamic abnormalities were corrected. One week after the procedure, the systolic blood flow velocity of the pulmonary artery and the diastolic blood flow velocity in the tricuspid valve were significantly reduced. The end-systolic length and width of the right atrium, the length and width of the right ventricle had decreased, these observations suggest the right ventricular volume load reducing gradually after the procedure. At 3 months and at 1 year after the procedure, various measures of the right heart system were reduced significantly in most patients compared with the preoperative data. All of these changes showed that the high-volume loading state of the right heart was reduced significantly.

Evaluating right ventricular (RV) function with 2-dimensional echocardiography is a troublesome endeavor. [31,32] RV-FAC and RVEF, which are methodologically easy to perform, may be a better echocardiographic descriptor of right ventricular systolic function than existing methods. The Tei index is a measurement of global myocardial function that has been used to evaluate both left ventricular and RV function. Derived from Doppler echocardiography, the Tei index is independent of ventricular geometry. The RV Tei index has been useful in patients with RV dysfunction and congenital heart disease and correlates well with RV ejection fraction in patients whose RV is their systemic ventricle. [10,11] Tricuspid annular plane excursion, which is easy to record, reflects RV function along the long axis, the predominant form of shortening, and relaxation of the RV free wall. The tricuspid annular plane systolic excursion (TAPSE) reflects the systolic RV function and has also been shown to be closely related to the RV ejection fraction. In this study, RV-FAC, RVEF, RV Tei index and TAPSE had significantly reduced compared the postoperative data with the preoperative data, which mean the right ventricular remodeling after device closure of an atrial septal defect gradually. These were mainly due to left to right shunt is blocked, right heart volume and pressure load rapidly decreased, and the right cardiac function improved partly. While the expanding right ventricle need some time to remodel, RV-FAC, RVEF and RV Tei index had reduced in the 3 month and 1 year’s follow-up, such change were not significant compared with 1 week’s follow-up. After a period of recover time, the cardiac function will reach a new equilibrium. [33]

Rigatelli and his colleagues assessed passive and active emptying of the LA, LA conduit function, and LA ejection fraction before and 6 months after the procedure, and then yearly. They found that relatively small occluder devices are probably effective enough to promote left atrial functional remodeling. [34] In the current study, gradual enlargement of the left atrium and the left ventricle was observed at 1 week, 3 months, and 1 year after the procedure. One year later, the structure and function of the left ventricle had normalized. Different measures of the left heart system had increased significantly at 3 months and at 1 year after the procedure, and the left ventricular ejection fraction had also increased significantly. These observations suggest that the increase observed in the load of left heart system capacity may have led to the left atrial and left ventricular enlargement. As the left-heart systolic function gradually improved to reach a normal level, left-heart function was maintained as right-heart function also recovered. In the current study, we tried to use small-sized occluders to ensure successful closure and to reduce the impact of the occluder on left atrial function. Left ventricular function is increased to varying degrees in patients with ASD, although the mechanism of this increase is not yet clear. Gao et al. reported that LV systolic function can be improved through normalization of IVS abnormal motion after transcatheter ASD occlusion, although transcatheter occlusion did not have a significant impact on intrinsic LV systolic synchronicity in patients with ASD. [35] The short axial view of the left ventricle after the procedure showed the restoration of heart structures with the left ventricle at the center, demonstrating that the previously abnormal geometric configuration of the left and right ventricles had been corrected.

The current study has a few shortcomings. First, the sample size is relatively small and the follow-up period is short. Therefore, studies with larger sample size and long-term follow-up should be carried out to verify our results. Because of financial limitations, our study includes relatively few indicators; later studies should include more qualitative and quantitative indicators. Second, we used relatively large occluders for only a few cases with relatively large ASDs. Although we have reported that the indications for transthoracic minimally invasive device ASD closure are broader than those for percutaneous ASD closure, and even that a 44-mm occluder can be used for some cases, we are not sure whether large occluders affect the intracardiac structures. Further large-sample follow-up studies should be performed to address this question. Third, the results of the current study are based on transthoracic minimally invasive device ASD closure. We do not know whether percutaneous ASD closure can achieve similar clinical outcomes, and would require further investigation to compare these procedures. Fourth, our study group included both adults and children. In further studies, we should evaluate pediatric and adult patients separately, especially when measuring changes in the size of the chambers of the heart.

In conclusion, transthoracic minimally invasive device ASD closure is safe and effective, as we have demonstrated in our previous studies.[2–4] Postoperative follow-up transthoracic echocardiography showed significantly improved heart structure and function compared with the preoperative data. Therefore, we recommend transthoracic device ASD closure as an alternative treatment for ASD.
